# Hop Polyphenols in Relation to Verticillium Wilt Resistance and Their Antifungal Activity

**DOI:** 10.3390/plants9101318

**Published:** 2020-10-06

**Authors:** Sabina Berne, Nataša Kovačević, Damijana Kastelec, Branka Javornik, Sebastjan Radišek

**Affiliations:** 1 Department of Agronomy, Biotechnical Faculty, University of Ljubljana, Jamnikarjeva 101, SI-1000 Ljubljana, Slovenia; sabina.berne@bf.uni-lj.si (S.B.); natasha.kovacevic@gmail.com (N.K.); damijana.kastelec@bf.uni-lj.si (D.K.); branka.javornik@bf.uni-lj.si (B.J.); 2 Slovenian Institute of Hop Research and Brewing, Cesta Žalskega tabora 2, SI-3310 Žalec, Slovenia

**Keywords:** *Humulus lupulus*, hop extract, plant disease resistance, antifungal, *p*-coumaric acid, tyrosol

## Abstract

(1) Background: Verticillium wilt (VW) of hop is a devastating disease caused by the soil-borne fungi *Verticillium nonalfalfae* and *Verticillium dahliae*. As suggested by quantitative trait locus (QTL) mapping and RNA-Seq analyses, the underlying molecular mechanisms of resistance in hop are complex, consisting of preformed and induced defense responses, including the synthesis of various phenolic compounds. (2) Methods: We determined the total polyphenolic content at two phenological stages in roots and stems of 14 hop varieties differing in VW resistance, examined the changes in the total polyphenols of VW resistant variety Wye Target (WT) and susceptible Celeia (CE) on infection with *V. nonalfalfae*, and assessed the antifungal activity of six commercial phenolic compounds and total polyphenolic extracts from roots and stems of VW resistant WT and susceptible CE on the growth of two different *V. nonalfalfae* hop pathotypes. (3) Results: Generally, total polyphenols were higher in roots than stems and increased with maturation of the hop. Before flowering, the majority of VW resistant varieties had a significantly higher content of total polyphenols in stems than susceptible varieties. At the symptomatic stage of VW disease, total polyphenols decreased in VW resistant WT and susceptible CE plants in both roots and stems. The antifungal activity of total polyphenolic extracts against *V. nonalfalfae* was higher in hop extracts from stems than those from roots. Among the tested phenolic compounds, only *p*-coumaric acid and tyrosol markedly restricted fungal growth. (4) Conclusions: Although the correlation between VW resistance and total polyphenols content is not straightforward, higher levels of total polyphenols in the stems of the majority of VW resistant hop varieties at early phenological stages probably contribute to fast and efficient activation of signaling pathways, leading to successful defense against *V. nonalfalfae* infection.

## 1. Introduction

Verticillium wilt (VW), caused by plant pathogenic fungi from the *Verticillium* sensu stricto genus, is one of the most serious soil-borne diseases, estimated to affect nearly 400 plant species, ranging from herbaceous annuals to woody perennials [1]. The colonization of host plants occurs through the root epidermis and, if successful, the fungus spreads to the vascular tissues, leading to systemic infection of the plant [2]. Although the disease symptoms vary among hosts, they typically include wilting, chlorosis, necrosis, and vascular discoloration [1,2,3]. The vascular plant pathogen *Verticillium nonalfalfae* [4] causes VW and plant death in several important crops [5]. VW of hop was first reported in 1924 in England [6], where mild and lethal (progressive) disease forms were described [7] and attributed to the pathogen virulence, the sensitivity of the hop cultivars, and ecological factors [8,9]. Genetic analysis and pathogenicity assays also confirmed mild and lethal groups among *V. nonalfalfae* hop isolates from Slovenia, United Kingdom, and Germany [10], and specific markers were developed to distinguish these two pathotypes [11]. The pathogenicity and the colonization studies revealed that the first foliar wilt symptoms appear approximately 20 days post inoculation (dpi) and rapidly progress in susceptible varieties, whereas resistant varieties (e.g., Wye Target) show no or mild symptoms [10,12]. Corresponding to the symptoms, the fungal biomass gradually increases in roots and stems of susceptible varieties, while in resistant plants, the colonization in roots is significantly less extensive, and fungal DNA in stems is barely detected [13,14]. As the diseased plant undergoes senescence, the fungus produces resting structures that are released in the soil, where they remain dormant for several years in the absence of a host [15]. In the past, soil fumigation was used to control VW effectively, but due to the toxicity and the detrimental effects on the environment, usage of these chemicals has been restricted or even banned [2]. Currently, disease management relies primarily on crop rotation and planting pathogen-free resistant cultivars, while other strategies, such as biofumigation, soil solarization, and soil amendments with biological control agents have had variable field-level efficacy and performed inconsistently [16].

On pathogen invasion, plants produce reactive oxygen species (ROS) [17] and mount a variety of defense responses [18], which can result in directly killing or inhibiting the pathogen, reinforcing local immune responses or priming cells in distal tissues [19]. In these processes, phenolics, chemically characterized as compounds with one or more benzene rings and at least one directly bonded hydroxyl group, which can be further methylated, methoxylated, aminated, or glycosylated, play significant roles [20]. Plant phenolics arise from the shikimate pathway (phenylpropanoids) or the acetate/malonate pathway (polyketides), or their combination (flavonoids), producing various monomeric (simple phenols) or polymeric structures (polyphenols) [21]. While constitutive phenolics are required for normal plant growth and development and can serve as signaling molecules and modulators of auxin IAA action, induced phenolics are secreted in response to physical injury, pathogen infection, or other stresses [20,22].

Xylem vessels, due to their structural design and transport function, provide a convenient avenue for rapid colonization of plants by vascular fungal pathogens. To fight off VW pathogens after their recognition by extracellular or intracellular receptors, plants mount various physical and chemical defense responses [23,24]. Among physical defenses, cell wall lignification is a particularly important early response, in which phenolics, released from the vacuoles of specialized phenolic-storing cells, are oxidized and polymerized with each other or cellular proteins and cell wall carbohydrates to produce lignified walls and/or suberized structures (with infusion of lipids into these complexes) [20]. Another common defense response to VW pathogens is the formation of tyloses, lateral outgrowths of paravascular parenchyma cells, following the Indole-3-Acetic Acid (IAA) build-up induced by oxidized phenolics [3,13,25]. Pectin-rich gels and gums are often accumulated around tyloses to prevent longitudinal spread of infection, while lateral colonization is inhibited by vascular coating and secondary cell wall and callose depositions [23]. Since complete sealing off of the vessels can result in drought stress, plants have adapted by activating a tissue-specific developmental program (vein clearing) that leads to the formation of new xylem elements [26]. In addition to physical defense responses, considerable metabolic changes occur in the parenchyma cells surrounding VW infected vessels. These include production of pathogenesis-related (PR) proteins, peroxidases and proteases in the apoplast and the xylem sap [27,28,29,30,31,32], as well as accumulation of various phenolic compounds [28,33,34,35,36,37,38].

Transcriptome studies of *V. dahliae* interactions with cotton [39,40] and tomato [30,41] have highlighted genes involved in the phenylpropanoid pathway and synthesis of lignin as upregulated and contributing to defense response in resistant plants. In contrast, our dynamic transcriptome profiling of *V. nonalfalfae* interactions with hop (*Humulus lupulus*) demonstrated biosynthesis of secondary metabolites and phenylpropanoid biosynthesis as the two processes enriched in the roots of susceptible plants [42]. The main objectives of this study were, therefore: (i) to assess the total polyphenols in roots and stems of VW resistant, moderately-resistant, and susceptible hop varieties sampled at two phenological stages, representing vegetative (before flowering, BF) and generative (mature cones, M) development phases; (ii) to explore the relationship between total polyphenols content and resistance response to *V. nonalfalfae* infection in susceptible variety Celeia (CE) and resistant Wye Target (WT); (iii) to study whether a single commercial phenolic compound can restrict *V. nonalfalfae* growth in vitro; and (iv) to investigate the potential antifungal activity of hop phenolic extracts on the growth of two different *V. nonalfalfae* hop pathotypes.

## 2. Results

### 2.1. Total Polyphenols Are Higher in Roots Than Stems and Increase With Phenological Stage in Different Hop Varieties

**Figure 1:**
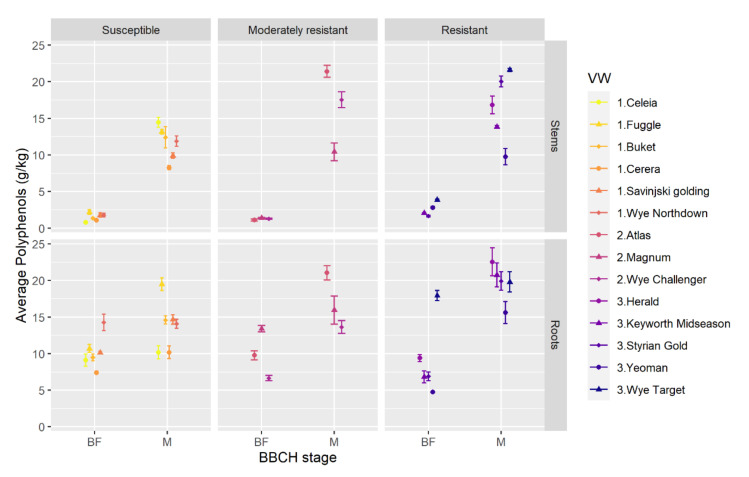
Average total polyphenols increase with phenological stage in different organs and hop varieties. Before flowering, hop roots have statistically significant higher average polyphenol content than stems, which is also statistically significant at the phenological stage of mature cones. Total polyphenols were determined spectrophotometrically according to the method adopted by the European Brewery Convention for determination of total polyphenols in beer [43]. Data are means ± SE (*n* = 3). BF, before flowering (BBCH stage 51–55); M, mature cones (BBCH stage 87–89), VW, Verticillium wilt resistance groups 1—susceptible, 2—moderately resistant, 3—resistant.

Total polyphenols were extracted from dry samples of hop roots and stems, collected at phenological (BBCH) stages 51–55 (before flowering, BF) and 87–89 (mature cones, M), and their concentration was determined spectrophotometrically [43]. Before flowering, the average total polyphenols in roots of all varieties were up to ten times higher than in stems. The highest values were determined in roots of VW resistant Wye Target, moderately resistant Magnum, and susceptible Wye Northdown (Figure 1). Among other varieties, there were no obvious differences that could link to VW resistance. At the mature cones stage, the content of total polyphenols in stems greatly increased in certain varieties reaching or even exceeding those in roots. In some varieties, the total polyphenols content in roots was comparable between the two phenological stages, while in others, it increased up to three times with maturation of the hops. Although no direct association between total polyphenols content and hop VW resistance groups could be confirmed, one of the most VW resistant varieties, Wye Target, had the highest level of polyphenols.

Analysis of variance revealed statistically significant triple interaction Variety: Organ: BBCH stage. Comparisons between average total polyphenols were therefore made for each individual hop variety, between roots and stems, at each BBCH stage separately (BF: roots-stems, M: roots-stems), and between the two BBCH stages, separately for roots and stems (roots: M-BF, stems: M-BF) (Table S1). Before flowering, the average total polyphenols in roots were statistically significantly higher than in stems in all hop varieties. At the mature phenological stage, the differences between average polyphenols in roots and stems were smaller and statistically significant only for Fuggle, Keyworth Midseason, Magnum, Savinjski Golding, and Yeoman. When comparing the two phenological stages, no statistically significant differences were found in the roots of Celeia, Cerera, Magnum, Savinjski Golding, Wye Northdown, and Wye Target. The average total polyphenols in the stems of all varieties were statistically significant higher at the mature stage than before flowering.

At the pre-flowering stage, the average total polyphenols in the roots of VW resistant variety Wye Target were statistically significantly higher than in other hop varieties, apart from Magnum and Wye Northdown (Table 1). In opposition, in the roots of VW resistant variety Yeoman, the average total polyphenols before flowering were statistically significantly lower than those of the VW susceptible varieties. In addition, the average total polyphenols in the roots of VW resistant Styrian Gold, Keyworth Midseason, and moderately resistant Wye Challenger were statistically significantly lower than in the VW susceptible Wye Northdown, Savinjski Golding, and Fuggle. At the mature stage, the average total polyphenols in the roots of VW susceptible varieties Cerera and Celeia were statistically significantly lower than those of the VW resistant and moderately resistant hop varieties. On the other hand, VW resistant Herald had statistically significantly lower average total polyphenols in the roots than the majority of VW susceptible varieties.

Before flowering, the stems of VW resistant varieties, apart from Herald and in certain cases Styrian Gold, had statistically significantly higher content of average polyphenols than the stems of VW moderately resistant and susceptible hop (Table 2). At the mature cones stage, the average total polyphenols in the stems of VW resistant and moderately resistant varieties, with the exception of Yeoman and Keyworth Midseason, were statistically significantly higher than in VW susceptible hop varieties.

The analysis of average total polyphenols in roots and stems of different hop varieties indicated that there is not a straightforward correlation between VW resistance and total polyphenol content. It is evident that total polyphenols increase in roots and stems with hop phenological stage and that before flowering hop roots have higher average total polyphenols than stems.

### 2.2. Total Polyphenols in Hop Decrease on VW Infection

The relationship between total polyphenols content and resistance response to *V. nonalfalfae* infection was tested in susceptible hop Celeia (CE) and resistant Wye Target (WT), selected due to the largest measured differences in the total polyphenols content. The plants were sampled 5 weeks after inoculation at the symptomatic stage of disease. The severe wilting symptoms only developed in susceptible variety CE, while plants of resistant WT appeared asymptomatic. Among *Verticillium*-inoculated plants, only those plants with confirmed pathogen re-isolation [44] were sampled for polyphenols analysis.

**Figure 2:**
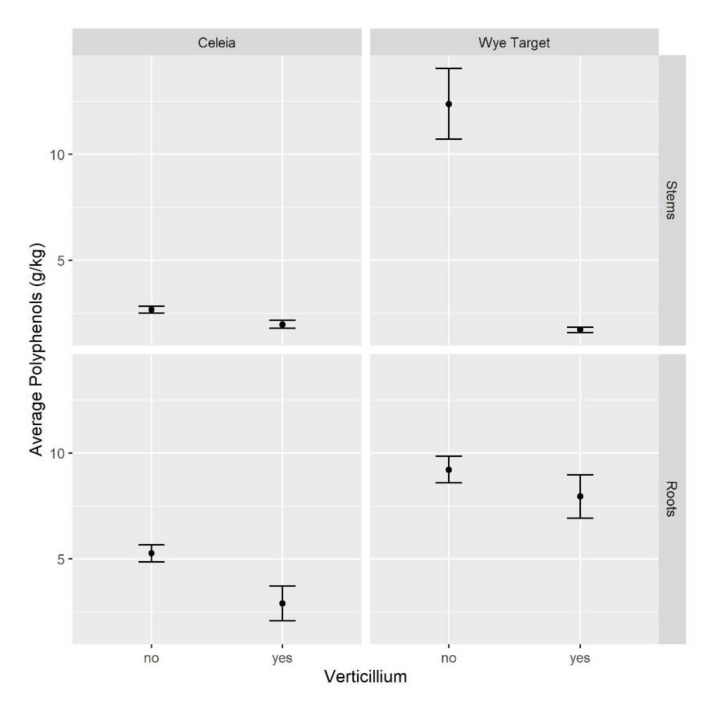
On VW infection, the average total polyphenols content decreases in susceptible hop Celeia and resistant hop Wye Target. Sampling of VW-infected (4 weeks after inoculation) and healthy plants was performed in triplicate at the leaf developmental stage (BBCH 17–19). Total polyphenols were determined spectrophotometrically as described in [43]. *Verticillium nonalfalfae* (isolate T2; lethal pathotype PV1) was used as the inoculum for root dipping [44]. Data are means ± SE (*n* = 3).

The average concentration of total polyphenols in healthy CE roots was 1.1–3.7 times higher than in infected plants at 95% confidence, while in stems, there were no statistically significant differences (Figure 2). On the other hand, the average total polyphenols in stems of healthy WT were 3.9–13 times higher than in those of infected plants, while no statistically significant differences were observed in roots.

### 2.3. p-Coumaric Acid and Tyrosol Inhibit V. nonalfalfae Growth In Vitro

**Figure 3:**
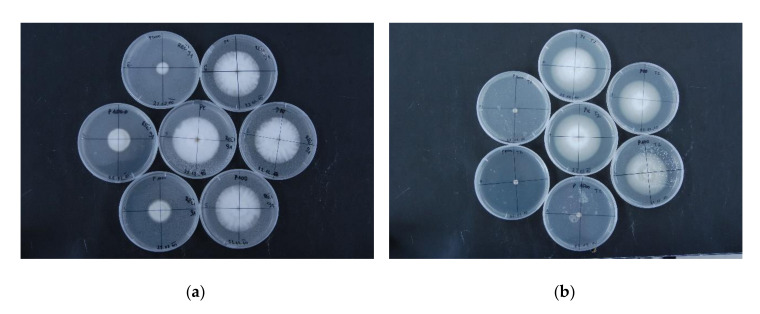
*V. nonalfalfae* growth inhibition on PDA supplemented with different concentrations of *p*-coumaric acid. Two isolates of *V. nonalfalfae* were selected for comparison: (**a**) Rec, mild pathotype M in the left panel and (**b**) T2, lethal pathotype PV1 in the right panel. Images were taken 14 days after mycelial disc inoculation on PDA plates supplemented with *p*-coumaric acid, dissolved in 20% methanol at six different concentrations (1, 10, 100, 1000, 1500, and 2000 ppm; depicted from top in a clockwise direction) or with solvent control (middle plate). All experiments were performed in six replicates.

Six commercial phenolic compounds were selected for evaluation of antifungal activity based on their inhibitory effects and accumulation in resistance response to *V. dahliae* infection of olive, tomato, and potato [30,37,38,45,46]. Two hop isolates of *V. nonalfalfae*, differing in their virulence [11], were tested: isolate—Rec (mild pathotype M) and highly virulent isolate T2 (lethal pathotype PV1) (Figure 3).

For compound comparison, the percentage inhibition of fungal growth for the highest tested concentration of individual phenolic compounds was calculated according to Equation (4), and results are listed in Table 3.

**Figure 4:**
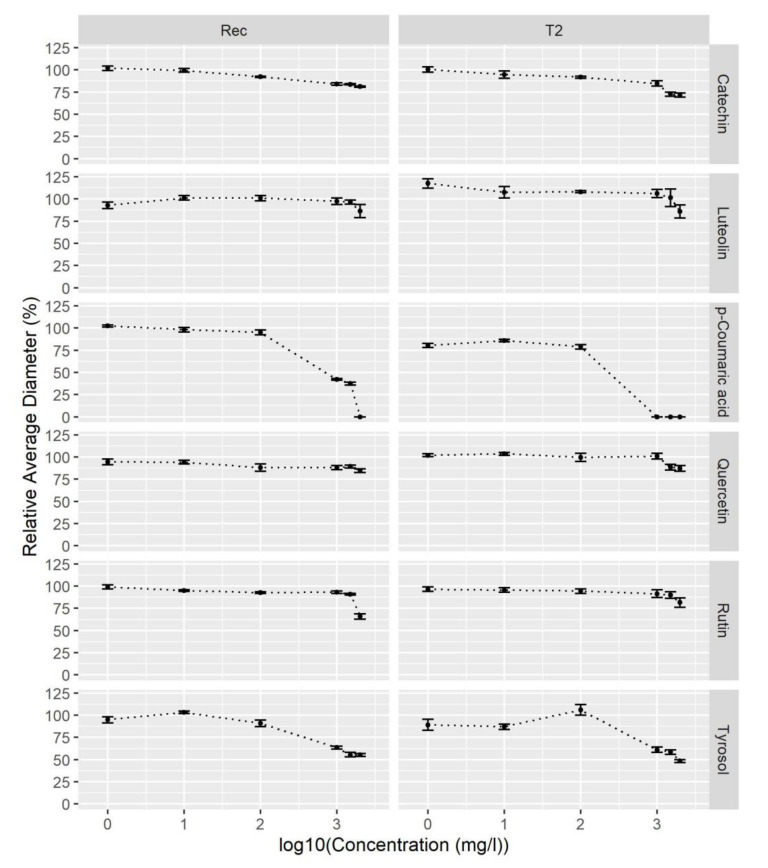
*p*-coumaric acid shows the highest inhibitory activity against *V. nonalfalfae*. The efficacies of six commercial phenolic compounds were expressed as the relative average diameter of fungal colonies (Equation (3) against the log10 of compound concentration). Rec, mild pathotype of *V. nonalfalfae*, T2, lethal pathotype of *V. nonalfalfae*. Data present the means ± SE (*n* = 6).

Catechin slightly inhibited fungal growth at concentrations higher than 100 mg/L and was more effective against lethal strain T2 (28% inhibition) than mild strain Rec (19% inhibition) at 2000 mg/L. Similar fungal growth inhibition (around 15%) was observed for luteolin and quercetin, with no differences between the fungal strains and rutin, which was more effective against Rec (34% inhibition) than T2 (18% inhibition) at 2000 mg/L. Tyrosol equally inhibited both strains, reaching around 50% fungal growth inhibition at 2000 mg/L. *p*-coumaric acid had the highest antifungal activity against *V. nonalfalfae* (Figure 4). No growth was observed at concentrations above 1000 mg/L for T2 and 2000 mg/L for Rec, respectively.

### 2.4. Total Phenolic Extracts from Hop Show Antifungal Activity against V. nonalfalfae

**Figure 5:**
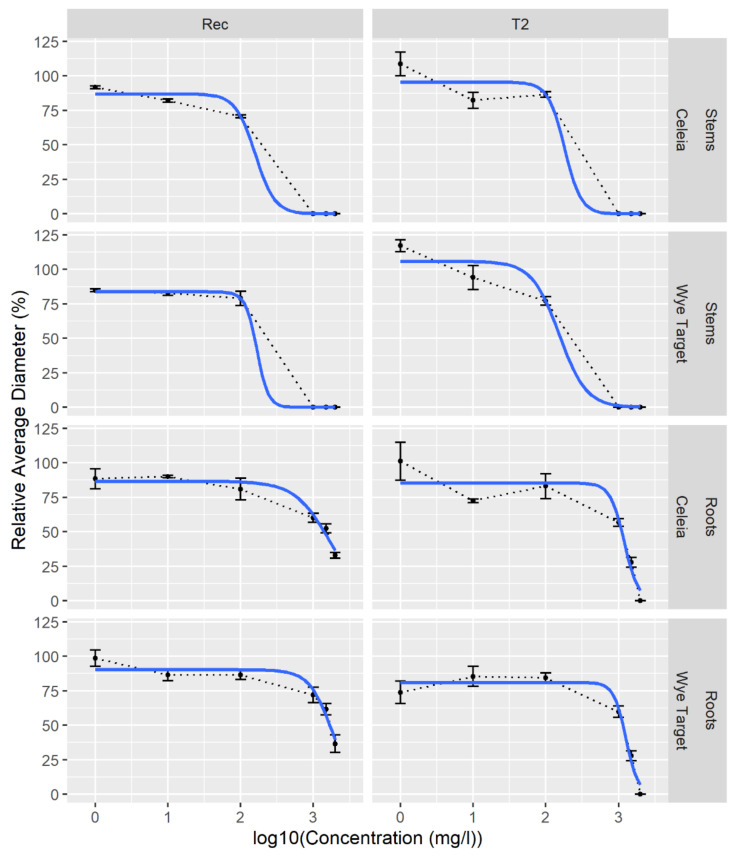
Polyphenolic extracts from hop roots and stems display antifungal activity against *V. nonalfalfae*. Using a nonlinear regression model, the relative average colony diameter (in % of solvent control) was analyzed depending on the logarithmic phenolic extract concentration. Rec, mild pathotype of *V. nonalfalfae*; T2, lethal pathotype of *V. nonalfalfae*; Celeia, VW susceptible hop; Wye Target, VW resistant hop.

Extracts of the total polyphenols from roots and stems of VW resistant WT and susceptible hop CE were screened against lethal (T2) and mild (Rec) pathotypes of *V. nonalfalfae* in order to correlate potential VW resistance with antifungal activity of hop polyphenols. Dose response curves (Figure 5) showed all hop extracts inhibited *V. nonalfalfae* growth. No fungal growth was observed at concentrations above 1000 mg/L of polyphenols extracted from hop stems. Lethal pathotype T2 was more susceptible to polyphenolic extracts from hop roots than mild pathotype Rec, since the latter continued growing even at 2000 mg/L of total polyphenols.

Estimated IC50 (concentration at which 50% inhibition of fungal growth was observed) values of polyphenolic hop extracts (Table 4) revealed that extracts from hop stems were around ten times more effective against *V. nonalfalfae* than extracts from roots, with minimal differences observed between the two hop varieties.

## 3. Discussion

### 3.1. Total Polyphenols in Roots and Stems of Different Hop Varieties

Phenolics from the lupulin glands of hop cones (hops) are well characterized, especially alpha and beta acids. They are traditionally used as a preservative and flavoring agent, adding bitterness and aroma to beer [47]. Pharmaceutical, cosmetic, and nutraceutical companies are attracted to the health-promoting properties of hops phenolics, particularly due to their antioxidant, anti-inflammatory, anticancer, anti-obesity, sedative, estrogenic, and anti-osteoporosis-related activities [48,49,50]. In addition, numerous antimicrobial activities of hops essential oils and crude leaf extracts are described (reviewed in [48,51]), including antibacterial effects towards Gram-positive and Gram-negative bacteria, inhibition of replication and proliferation of certain viruses, and potent activity of some prenylated chalcone derivatives against malaria-causing *Plasmodium falciparum*. Hops phenolics also display antifungal activity against *Trichophyton mentagrophytes* and *T. rubrum*, weak inhibition of *Fusarium oxysporum* and *Zymoseptoria tritici*, and very low activity against *Candida albicans*, *C. glabarta*, and some *Pencillium* and *Aspergillus* species [48,51,52].

On the other hand, only a few studies have reported on phenolics from hop leaves, stems, and roots [52,53,54,55], especially in relation to their antifungal activity [52]. Here, we quantified the total polyphenols in roots and stems of 14 hop varieties before flowering and at the mature hops stage. We show that total polyphenols vary between different hop varieties (Figure 1) but are, in general, significantly higher in roots than stems in the pre-flowering stage, while the content of total polyphenols with hop maturation increases, especially in stems, reaching levels comparable to those in roots. Observed variation among hop varieties corroborates studies of hops phenolics, which report that the chemical composition of hops is specific to an individual hop variety and influenced by seasonal, climatic, and edaphic variation, the maturation stage of hop cones, and exposure to biotic stresses [53,55,56,57,58]. Comparison of hop phenolics, however, is difficult due to different approaches used for their determination, including sample pre-treatment, use of different solvents, extraction protocols, and analytical techniques, as well as expression of a compound’s abundance in different units [59].

We report that, before flowering, the total polyphenols in the roots of VW resistant variety Wye Target are significantly higher than in other hop varieties, while the rest of VW resistant and moderately resistant varieties at this stage have comparable or often even lower total polyphenols content in roots than VW susceptible varieties (Table 1, top triangle). In contrast, the total polyphenols in the stems of VW resistant varieties, except Herald, are significantly higher relative to VW moderately resistant and susceptible hop (Table 2, top triangle). At the mature cones stage, the total polyphenols in the roots of VW resistant and moderately resistant hop varieties are higher than in VW susceptible varieties Cerera and Celeia (Table 1, bottom triangle). In addition, total polyphenols in the stems of the majority of VW resistant and moderately resistant hop varieties, except Yeoman and Keyworth Midseason, are higher than in VW susceptible varieties (Table 2, bottom triangle). Our findings thus suggest that the relationship between total polyphenols and VW resistance is not explicit, although higher levels of phenolics, particularly in the stems of the majority of resistant and moderately resistant hop varieties at early and mature phenological stages, may contribute to observed differences in the resistance responses to *V. nonalfalfae*. It has been determined that many VW infections stay in the pre-vascular phase of the disease [60,61], with phenolic compounds accumulating in roots and stems of resistant as well as susceptible plants, but the rate and the level of their production is higher in resistant plants [3]. Our recent study monitoring the changes in hop phenolic compounds induced on *V. nonalfalfae* infection corroborates these findings [14].

### 3.2. Phenolic Compounds Are Involved in the Plant Defense against VW Infection, but the Responses Vary among Host Species and with Disease Progression

Breeding and growing disease resistant lineages is essential in confronting VW pathogens. In tomato, a single major gene, the *Ve1* gene [62], confers resistance to *V. dahliae* race 1 strains, expressing Ave1 effector [63]. Similar resistance responses have been observed within and outside the Solanaceae family for various *Ve1* homologs [64]. However, several studies have reported that VW resistance is controlled by additive quantitative genetic components located in multiple small effect genomic loci (quantitative trait locus, QTLs) [65,66,67,68,69,70,71]. Among these, the QTL for *V. longisporum* resistance in oilseed rape co-localized with QTLs for a number of phenylpropanoids [72]. In addition, phenolic acids and soluble phenylpropanoid metabolites have been implicated in Arabidopsis resistance response to *V. longisporum* [33,34] and in potato response to *V. dahliae* [46]. Accumulation in phenolic and lignin content has also been described in broccoli and cauliflower as a result of *V. dahliae* infection, but with decisive differences in the speed of lignification and phenolics accumulation [73]. In a susceptible olive cultivar, the total phenolics increased on *V. dahliae* infection [38,74] and as a defense reaction to secreted fungal phytotoxins [75]. Following *V. nonalfalfae* infection, the genes involved in the biosynthesis of phenylpropanoids were induced in VW susceptible hop [42]. However, in the *V. dahliae* interaction with cotton [40] and tomato [30], upregulation of genes in the phenylpropanoid metabolism and synthesis of lignin contributed to effective defense response in resistant plants. In the pre-vascular stage of infection, accumulation of flavanols in the roots of resistant cotton on inoculation with *V. dahliae* was detected [76]. A consistent response was observed at the asymptomatic stage of VW disease in the roots and the stems of resistant hop after exposure to *V. nonalfalfae*, although a significant increase in total flavanols was also found in the stems of susceptible hop 3 days after inoculation [14].

In this study, we assessed total polyphenols at the symptomatic stage of VW disease, 5 weeks after *V. nonalfalfae* inoculation, when susceptible plants exhibited severe symptoms. We deliberately chose VW susceptible Celeia, which had the lowest measured content of total polyphenols, and resistant Wye Target, which had the highest total polyphenols among all the tested varieties. A statistically significant decrease in total polyphenols was observed in the roots of susceptible hop, as was an even more prominent drop in the stems of resistant hop relative to healthy plants. Our results confirm recent findings by Kunej et al. [14], who reported that, at the late stages of infection, flavanols and hydroxycinnamic acids decreased at 15 and 6 days after inoculation in susceptible and resistant hop, respectively. A similar decrease in total phenol content has also been reported in resistant and susceptible olive cultivars on infection with *V. dahliae* [37].

There are several possible explanations for the noted decrease in total polyphenols. First, the fungus can actively suppress plant defense by secreting various hydrolytic enzymes and effector proteins, interfering with the host metabolism [77]. For example, a secreted lignin-modifying peroxidase from *V. nonalfalfae*, VnaPRX1.1277, has been implicated in protection against plant-derived oxidative stress and/or degradation of lignin [78]. It is highly expressed in the stems of VW susceptible hop, particularly at the later stages of infection, and may contribute towards the observed drop in total polyphenols. In addition, some strains of *V. dahliae* are able to degrade and utilize ortho-phenolic compounds as a source of carbon [61].

Another reason for the decline in total polyphenols may be due to their diversion to other products, as suggested for the *V. dahliae*–olive interaction [37]. There is strong evidence that the lignin metabolism is central to the VW resistance response, as has been shown in *V. dahliae* infection of cotton [40], broccoli, cauliflower [73], tomato [30], and pepper [79], in *Brassica napus* [80] and *Arabidopsis thaliana* [34] as a defense response to *V. longisporum*, and in the *V. nonalflafae* interaction with hop [13]. Notably, there are clear differences in the rate and the extent of lignin synthesis and deposition among resistant and susceptible cultivars, with earlier onset of the lignification process and a stronger reaction in the resistant cultivar, indicating the importance of pathway activation in a timely and efficient manner [30,40,81]. In respect to esterification and crosslinking of polyphenols to the plant cell wall components during the lignification process, we cannot exclude the possibility that the decrease in total polyphenols could partially result from a technical flaw, since bound phenolics are more difficult to extract and usually remain in the plant residues [82].

### 3.3. V. nonalfalfae Growth In Vitro Is Inhibited by p-Coumaric Acid and Tyrosol

Hop polyphenols are a complex mixture of phenolic acids and non-prenylated flavonoids, including proanthocyanidins and flavonol glycosides [47]. Some of these compounds have been shown to accumulate in the defense response of host plants after infection with *Verticillium* [14,28,37,46,83,84,85], and for certain compounds, antifungal activity has been tested in vitro [38]. 

Flavanol catechin is known to have antifungal activity against root-colonizing fungi [86] and inhibit sporulation [61], but its activity against *V. dahliae* (IC_50_ = 2100 mg/L) is low. Similarly, we observed very weak inhibition of *V. nonalfalfae*; at a catechin concentration of 2000 mg/L, the fungal growth was 82% and 72% that of the control for mild strain Rec and lethal strain T2, respectively.

Flavonol quercetin and its glycoside form rutin, which, at 2000 mg/L, inhibited around 15% of T2 fungal growth, while rutin was more effective against mild strain Rec, inhibiting 34% of fungal growth. In contrast to these findings, in the *V. dahliae*–olive interaction, quercetin displayed high antifungal activity against *V. dahliae* with IC_50_ of 6 mg/L, while rutin was 13 times less active [38]. In potato, *V. dahliae* induced accumulation of antifungal rutin [45]; however, the fungus was able to metabolize this flavonol glycoside, using glucosidases and rhamnosidases, into aglycone quercetin, which was further detoxified by quercetinases to release phloroglucinol and a by-product, protocatechuic acid [87]. In a proposed hypothetical model, the latter may be converted to salicylic acid (SA) to elicit the SA signaling pathway and counter the jasmonate signaling, ultimately resulting in plants more susceptible to this disease [87].

A plant flavone luteolin, another compound that accumulates in olive after *V. dahliae* infection, had high antifungal activity against *V. dahliae* with IC_50_ of 7 mg/L [38]. In addition to inhibiting fungal growth, luteolin and quercetin caused ultrastructural and morphological changes in the fungus, including increased thickness of the fungal cell wall, increased number of vesicles with cell wall deposition material, and a higher number of mitochondria, which also doubled their size. Furthermore, sporulation was reduced, and conidia that were produced did not germinate [38]. In contrast to relatively good inhibition of *V. dahliae*, luteolin exhibited poor antifungal activity against *V. nonalfalfae* strains (fungal growth was 85% that of the control).

Phenyletanoid tyrosol, a potent antioxidant [88] and one of the main phenolic compounds found in virgin olive oil, decreased in response to *V. dahliae* infection [89] and had medium antifungal activity (IC_50_ = 660 mg/L) [38]. Its antifungal activity against *V. nonalfalfae* was good, achieving 50% growth inhibition of both lethal and mild strains.

*p*-coumaric acid possesses broad and strong antibacterial activity [90]. Together with ferulic acids, it is found in xylan and is responsible for esterification of lignin and strengthening the plant cell wall [91]. Although *p*-coumaric acid increased on *V. dahliae* infection in potato [45] and tomato [30], it had low inhibitory activity (IC_50_ = 1470 mg/L) [38]. Quite the opposite was found for *V. nonalfalfae*, whose growth was completely inhibited by *p*-coumaric acid at concentrations above 1000 mg/L for T2 and 2000 mg/L for Rec.

In the present study, we did not measure the concentrations of individual phenolic compounds in hop samples, and we could not find studies reporting the concentrations of *p*-coumaric acid in stems and roots of hop. However, several research groups conducted HPLC analysis of *p*-coumaric acid in methanol, water, or ethanol extracts from hop cones and leaves [92,93,94]. The values for hop cones ranged from 6 μg/g dry matter to 256 μg/g dry matter, depending on the hop variety and the extraction solvent used, while the concentration of *p*-coumaric acid in leaves was 45 μg/g dry matter. We conclude that, in physiological conditions, *p*-coumaric acid does not reach high enough concentrations to inhibit *V. nonalfalfae* growth, the effect observed in the antifungal experiments in vitro.

### 3.4. Antifungal Activity of Total Polyphenol Extracts from Hop

Although hops phenolic compounds have high and broad antibacterial activity [48], their antifungal activity is reportedly weak. A water extract from hop cones exhibited discrete (>20%) antifungal activity against certain *Aspergillus* and *Penicillium* species that were considered responsible for spoilage of bakery goods [95]. Crude ethyl acetate, acetone, and methanol extracts from the spent hops displayed antifungal activity against *Fusarium oxysporum*, *F. culmorum,* and *F. semitectum,* with the lowest MIC50 of 0.5 mg/mL, while methylene chloride extract exerted antifungal activity against *Botrytis cinerea* with an MIC50 of 1 mg/mL [96]. Column chromatography purified hops methanol extracts were effective against the human pathogenic fungi of *Trychophyton* spp., with isopentyl derivatives of humulone showing MIC between 12.5 and 50 μg/mL, while 3-isopentenylphlorisovalerophenone was also active against *Mucor rouxianus*, *Candida albicans*, and *Fusarium oxysporum*, exhibiting MICs similar to those of griseofulvin [97].

Compared to hops essential oil (IC_50_ = 0.36 g/L; 100% inhibition) or crude hops extracts (IC_50_ = 0.73 g/L; 85% inhibition), the inhibitory activity of methanol extracts from hop leaves, stems, and roots was relatively low (25–35% inhibition) when assessed against the wheat pathogen *Zymoseptoria tritici* [52]. In our study, dose response curves of methanol extracts from hop roots and stems revealed that the total polyphenols from stems (IC_50_ of 130–211 mg/L) were ten times more active against *V. nonalfalfae* than those from roots (IC_50_ of 1171–1804 mg/L). Furthermore, extracts from VW susceptible CE stems were more effective against mild strain Rec than lethal strain T2, while VW resistant WT stem extracts had the opposite effect on the fungal strains, with T2 being more sensitive. 

In summary, the results of the present study indicate that total polyphenols content exhibits great variability among different hop varieties but is generally higher in roots than stems and increases with hop maturation. Although the correlation between VW resistance and total polyphenol content is not straightforward, it is likely that phenolic compounds are involved in hop defense against *V. nonalfalfae* infection. The higher levels of total polyphenols, particularly in the stems of the majority of VW resistant hop varieties, probably contribute to fast and efficient activation of signaling pathways and defense responses, while these processes may be delayed in susceptible plants. Total polyphenols extracted from hop roots and stems are able to inhibit *V. nonalfalfae* growth, with clear differences in the sensitivity of fungal strains that may be attributed to a different composition of phenolic compounds in Celeia and Wye Target. Even though *p*-coumaric acid completely abolishes *V. nonalfalfae* growth in vitro, the concentrations necessary for such inhibition are not physiologically relevant.

## 4. Materials and Methods 

### 4.1. Plant Material

All plant material was collected in variety collection field SN5 at the Slovenian Institute of Hop Research and Brewing. Sampling of 14 varieties of hop (*Humulus lupulus* L.) differing in resistance to VW [98,99,100] (Table 5) was performed at two BBCH phenological growth stages of hop [101]: first before flowering (39–51) and second at maturation of hop cones (86–88). Nine plants of each variety were divided into triplicates containing three samples, separately for the bottom 1.5 m part of the bines without leaves and for the roots.

### 4.2. Artificial Inoculation of Hop with Verticillium nonalfalfae

*Verticillium* isolates designated T2 (lethal pathotype PV1) and Rec (mild pathotype M) were obtained from the culture collection of the Slovenian Institute of Hop Research and Brewing fungal collection. Fungi were maintained at 24 °C in the dark on potato dextrose agar (PDA) plates (Fluka Analytical, Buchs, Switzerland).

Artificial inoculation of hop was performed with root dipping in a *V. nonalfalfae* conidia suspension [44]. Sampling of VW-inoculated and mock-inoculated plants of VW susceptible Celeia and resistant Wye Target was performed 5 weeks after inoculation, when plants of Celeia had developed severe wilt symptoms. Root and stem samples from *Verticillium* inoculated plants were taken only from plants with confirmed pathogen re-isolation [44].

### 4.3. Extraction of Total Polyphenols from Dry Plant Material

Plant material was dried at 45 °C for 3 days, milled in a laboratory blender and weighed. The moisture content was then determined as:

(1) $$W\;=\;\frac{\left(m_{1}{\;-\;m}_{2}\right)}{\left(m_{1}{\;-\;m}_{0}\right)}\text{× }100$$

where W is the percent of moisture, m_0_ is the mass of a dish, m_1_ is the mass of a dish with sample prior to drying, and m_2_ is the mass of a dish with sample after drying. For all hop varieties, a moisture content was determined between 68 and 72%.

The extraction of total polyphenols was performed in two steps. First, 1 g of dry plant material was extracted for 30 min on a rotary shaker with 50 mL of 25% N,N-dimethylformamide (*v*/*v*, Sigma Aldrich Chemie GmbH, Germany) and filtered. Filtrate (25 mL) was then extracted with 25 mL of chloroform (Sigma Aldrich, Chemie GmbH, Germany) in a funnel separator. The top aqueous phase was transferred to a distillation flask, and chloroform was removed with a Büchi rotavapor R-200 (Flawil, Switzerland) at 45 °C. The remaining solution was transferred to a measuring flask and filled up to 50 mL with distilled water to obtain solution A.

### 4.4. Spectrophotometric Determination of Hop Total Polyphenols

Total polyphenols were analyzed according to the method adopted by the European Brewery Convention for determination of total polyphenols in beer [43]. Solution A (10 mL) was mixed with 8 mL of carboxymethyl celullose (CMC)/ ethylenediaminetetraacetic acid (EDTA) solution (Fluka Analytical, Buchs, Switzerland, 1% CMC (*m*/*v*)/0.2% EDTA (*m*/*v*)), 0.5 mL of 3.5% ammonium iron(III) citrate solution (Fluka Analytical, Buchs, Switzerland), and 0.5 mL of ammonia solution (Sigma Aldrich Chemie GmbH, Germany, concentrated ammonia diluted with two volumes of distilled water) and filled up to 25 mL with distilled water. After mixing and 10 min incubation at room temperature, the absorbance of total polyphe6nols was measured on an HP 8453 spectrophotometer (Hewlett Packard, California, USA) at 600 nm. For a blank, solution A was mixed with CMC/EDTA and ammonia solutions without the ferric reagent. The content of total polyphenols was calculated according to the European Brewery Convention (Analytica EBC, Method 7.14, [102]):

(2) $$P\;=\;\frac{(A\text{ × }820)\;}{\left(W\text{ × }20\right)}$$

where P is the polyphenol content in %, A is the absorbance measured at 600 nm (sample—blank sample), 820 is the standard coefficient [102], and W is the sample weight in g.

### 4.5. Inhibition of Fungal Growth by Commercial Phenolic Compounds

Based on results from the *Verticillium dahliae*–olive pathosystem, six commercial phenolic compounds were selected for assessment of antifungal activity against *V. nonalfalfae* strains T2 and Rečica. Mycelial discs (5 mm) taken from a 12–20-day old fungal culture were centrally inoculated on potato dextrose agar (PDA) plates supplemented with phenolic compound (quercetin, *p*-coumaric acid, catechin, rutin, tyrosol, and luteolin; all from Sigma Aldrich, Chemie GmbH, Germany) dissolved in 20% methanol at six different concentrations (1, 10, 100, 1000, 1500, and 2000 ppm) or with solvent control. Each compound was tested at each concentration in six replicates. Fungi were grown for 21 days in the dark at 20 °C and 60% humidity. Every seven days, fungal growth was measured as the diameter of the fungal colony minus the size of the mycelial disc along two perpendicular lines drawn across the middle of the mycelial disc. Relative average diameters (RAD) of fungal colonies were calculated as:

(3) $$\mathrm{RAD}\;(\%)\;=\;\frac{T}{C}\times 100\;$$

in which C is the average colony diameter in the control, and T is the average colony diameter in the treatment. For the highest tested compound concentration, percentage inhibition was also calculated as:

(4) $$\mathrm{Inhibition}\;(\%)\;=\;\frac{C-\;T}{C}\times 100\;$$

### 4.6. Determination of Antifungal Activity of Hop Polyphenolic Extracts

Hop extracts were prepared from combined samples of roots and combined samples of stems. The average content of polyphenols determined spectrophotometrically for these combined samples was 9.93 g/kg for Celeia roots, 11.63 g/kg for Celeia stems, 18.61 g/kg for Wye Target roots, and 20.61 g/kg for Wye Target stems. These values are a bit lower than the values reported for the total polyphenols in hop cones [93], where the total polyphenols comprise up to 4% of the total weight of dried hop cones [103].

Dried hop samples were extracted with 75% acetone (Fluka Analytical, Buchs, Switzerland; 50 mL per 1 g of plant material) for 30 min on a shaker at 150 rpm. Samples were filtered, and 50 mL of filtrate was extracted with 20 mL of chloroform (Sigma Aldrich Chemie GmbH, Germany) by vigorous shaking for 1 min followed by 20 min centrifugation at 3000 rpm in a Heraeus centrifuge Biofuge primo. The supernatant was transferred in a funnel separator, and the top aqueous phase was collected in a distillation flask. Chloroform was removed on a Büchi rotavapor R-200 (Flawil, Switzerland) at 45 °C. Remaining dark brown resin was dissolved in methanol at 200 mg/mL and diluted with distilled water to 20 mg/mL. The mass of extracted polyphenols was calculated from the concentration of polyphenols determined spectrophotometrically and the mass of the dry hop sample. From this mass, 10% was subtracted based on the assumption that there was such a loss during the extraction procedure.

The antifungal activity of hop polyphenolic extracts against *V. nonalfalfae* strains T2 and Rečica was determined on PDA plates supplemented with 1, 10, 100, 1000, 1500, and 2000 ppm of extracted polyphenolic compounds. Mycelial discs (5 mm), taken from a 12–20-day old fungal culture, were centrally inoculated and incubated in the dark at 20 °C and 60% humidity for three weeks. Fungal growth was measured at weekly intervals as the diameter of the fungal colony minus the size of the mycelial disc along two perpendicular lines drawn across the middle of the mycelial disc.

### 4.7. Statistical Analysis

All statistical analyses were performed with the R program [104]. In the first experiment, the data of total polyphenols were analyzed on the basis of a linear model that considered fixed effects of the variety (14 different varieties, Table 4), the BBCH stage (PB, M), the organ (stems, roots), three double interactions (variety:BBCH stage, variety:organ, BBCH stage:organ), and one triple interaction (variety:BBCH stage:organ). The dependent variable total polyphenols were log-transformed based on variance homogeneity assumptions. The mean values of log (total polyphenols) between the selected treatments were compared based on a simultaneous null hypothesis test using the “*glht*” function from the multcomp package in R. A similar statistical analysis was performed for the second experiment, in which the fixed effects were determined with variety, organ, and *Verticillium* infection. In the fourth experiment, the relative diameter was modeled with a nonlinear model using the “*nls*” function from the nlme package in R. The sigmoid curve of the dose–response relationship was determined according to:

(5) $$RAD=\phi_{1}{\left(1+e^{\left(\frac{\phi_{2}-log_{10}x}{\phi_{3}}\right)}\right)}^{-1}$$

where RAD is the relative colony diameter; ϕ_1_ is the upper asymptote; ϕ_2_, is log 10 of the concentration (x) at which a bend is reached at ϕ_1_/2; and ϕ_3_ is the rate of decrease of the relative diameter after the bend. All three parameters were compared between treatments based on their asymptotic confidence intervals. For hop extracts comparison, IC_50_, the concentration at which 50% inhibition of fungal growth is observed, was calculated from:

(6) $$IC50=10^{\phi_{2}}$$

## Figures and Tables

**Table 1:** Comparison of average total polyphenols in hop roots before flowering (BBCH stage 51–55; table—top triangle above grey-boxed area) and at the mature cones stage (BBCH stage 87–89; table—bottom triangle below grey-boxed area).

VW	Variety	WT	StG	KM	Y	H	WC	A	M	WN	Cer	SG	B	F	Cel
1	WT		***	***	***	***	***	***			***	***	***	***	***
1	StG								***	***		*		**	
1	KM								***	***		*			
1	Y					***		***	***	***	**	***	***	***	***
1	H									*					
2	WC			*		**		*	***	***		*		**	
2	A						*								
2	M										***			**	*
3	WN			*		**		*			***		*		**
3	Cer	***	***	***	*	***		***	**						
3	SG					*									
3	B					*									
3	F										***				
3	Cel	***	***	***	*	***		***	**					***	

VW resistance group: **1**, resistant; **2**, moderately resistant; **3**, susceptible; hop varieties: WT, Wye Target; StG, Styrian Gold; KM, Keyworth Midseason; Y, Yeoman; H, Herald; WC, Wye Challenger; A, Atlas; M, Magnum; Wye Northdown; Cer, Cerera; SG, Savinjski Golding; B, Buket; F, Fuggle; Cel, Celeia. Statistical significance based on contrast analysis for a linear model considering simultaneous multiple null hypothesis testing: *, *p* < 0.05; **, *p* < 0.01; ***, *p* < 0.001; blue squares, significantly lower average total polyphenols in the variety specified in row compared to the variety specified in column; orange squares, significantly higher average total polyphenols in the variety specified in row compared to the variety specified in column.

**Table 2:** Comparisons of average total polyphenols in hop stems before flowering (BBCH stage 51–55; table—top triangle above grey-boxed area) and at the mature cones stage (BBCH stage 87–89; table—bottom triangle below grey—boxed area).

VW	Variety	WT	StG	KM	Y	H	WC	A	M	WN	Cer	SG	B	F	Cel
1	WT		***	***		***	***	***	***	***	***	***	***	***	***
1	StG				***			*			*				***
1	KM	**				***	**	***	*		***		*		***
1	Y	***	***			***	***	***	***	**	***	**	***		***
1	H				***					*		*		***	
2	WC				***									***	***
2	A			**	***					**		**		***	
2	M	***	***			**	***	***						**	***
3	WN	***	***				*	***			**				***
3	Cer	***	***	***		***	***	***				**		***	
3	SG	***	***			***	***	***							***
3	B	***	**					***			*			**	***
3	F	***	*					**			**				***
3	Cel	*			*			*			***				

VW resistance: **1**, resistant; **2**, moderately resistant; **3**, susceptible; WT, Wye Target; StG, Styrian Gold; KM, Keyworth Midseason; Y, Yeoman; H, Herald; WC, Wye Challenger; A, Atlas; M, Magnum; Wye Northdown; Cer, Cerera; SG, Savinjski Golding; B, Buket; F, Fuggle; Cel, Celeia. Statistical significance based on contrast analysis for a linear model considering simultaneous multiple null hypothesis testing: *, *p* < 0.05; **, *p* < 0.01; ***, *p* < 0.001; blue squares, significantly lower average total polyphenols in the variety specified in row compared to the variety specified in column; orange squares, significantly higher average total polyphenols in the variety specified in row compared to the variety specified in column.

**Table 3:** Percentage inhibition of fungal growth two weeks after *V. nonalfalfae* inoculation on PDA plates supplemented with phenolic compound.

Fungal Strain	Catechin	Luteolin	*p*-Coumaric Acid	Quercetin	Rutin	Tyrosol
Rec	18.9 ± 0.8	13.7 ± 7.3	100 ± 0	15.3 ± 2.1	34.2 ± 3.1	44.8 ± 1.6
T2	28.2 ± 2.3	14.0 ± 7.3	100 ± 0	12.9 ± 3.2	18.4 ± 5.3	51.7 ± 1.7

Data represent means ± SE (*n* = 6) of colony diameters at the highest compound concentration of 2000 mg/L. Rec, mild pathotype of *V. nonalfalfae*, T2, lethal pathotype of *V. nonalfalfae* [11]. C, average colony diameter in control; T, average colony diameter in treatment.

**Table 4:** Estimated IC_50_ values of polyphenolic extracts with 95% confidence intervals (CI) from hop roots and stems against *V. nonalfalfae in vitro* growth.

Extract	Strain	ϕ_1_, asymptote	IC_50_ (mg/L)
CE stems	Rec	87 (CI 79−95)	148 (CI 121−246)
	T2	96 (CI 88−103)	183 (CI 137−530)
WT stems	Rec	84 (CI 76−91)	212 (CI 141−604)
	T2	106 (CI 98−113)	130 (CI 113−163)
CE roots	Rec	84 (CI 77−90)	1683 (CI 1485−1911)
	T2	86 (CI 80−92)	1172 (CI 1041−1308)
WT roots	Rec	89 (CI 83−95)	1804 (CI 1617−2032)
	T2	82 (CI 76−88)	1223 (CI 1088−1365)

Dose response sigmoid curves were defined by three parameters: ϕ_1_, upper asymptote; ϕ_2_, log 10 of concentration at which a bend is achieved at ϕ_1_/2; and ϕ_3_, the rate of decrease of the relative diameter after the bend. Analysis showed that the same value (−0.12, CI −0.15 to −0.09) can be attributed to ϕ_3_, while parameters ϕ_1_ and ϕ_2_ varied between treatments. For ϕ_2_, antilog values are listed and represent estimated IC50 with corresponding 95% confidence intervals (CI). Rec, mild *V. nonalfalfae* strain Rečica, T2, lethal *V. nonalfalfae* strain [11].

**Table 5:** List of hop varieties differing in resistance to Verticillium wilt (VW).

Hop Variety	VW Resistance
Wye Target	resistant
Styrian Gold	resistant
Keyworth Midseason	resistant
Yeoman	resistant
Herald	resistant
Wye Challenger	moderately resistant
Atlas	moderately resistant
Magnum	moderately resistant
Wye Northdown	susceptible
Cerera	susceptible
Savinjski Golding	susceptible
Buket	susceptible
Fuggle	susceptible
Celeia	susceptible

## Supplementary Materials

The following table is available online at https://www.mdpi.com/2223-7747/9/10/1318/s1, Table S1: Comparisons between average total polyphenols for each individual hop variety between roots and stems at each BBCH stage separately.
